# Trends of cannabis use and related harms before and after legalization for recreational purpose in a developing country in Asia

**DOI:** 10.1186/s12889-023-15883-6

**Published:** 2023-05-19

**Authors:** Rasmon Kalayasiri, Suriyan Boonthae

**Affiliations:** 1 Department of Psychiatry, Epidemiology of Psychiatric Disorders and Mental Health Research Unit, Faculty of Medicine, Chulalongkorn University, Pathumwan, Bangkok, 10330 Thailand; 2 Department of Psychiatry, King Chulalongkorn Memorial Hospital, Pathumwan, Bangkok, 10330 Thailand; 3 Research Centre for Social and Business Development, Chatuchak, Bangkok, 10900 Thailand

**Keywords:** Cannabis, Legalization, Substance, Laws, Prevalence, Harm

## Abstract

**Background:**

Thailand was the first country in Asia to legalize the use and purchase of cannabis leaves in February 2021 and the whole plant in June 2022 after the 2019 allowance for medical purposes. The study explored trends in cannabis use in Thailand before and after the recreational cannabis allowance was imposed.

**Methods:**

Cannabis and other variables of substance use, cannabis use disorder, and attitude towards cannabis of the Thai population aged 18 to 65 years in 2019 (n = 5,002), 2020 (n = 5,389) and 2021 (n = 5,669) were obtained from annual surveys conducted in the last two months of each year by the Centre for Addiction Studies. The surveys were repeat cross-sectional surveys of the general population of Thailand. Repeated variables from at least two annual surveys were included for analysis using the Chi-square test and the t-test.

**Results:**

The prevalence of cannabis use in the past year had increased from 2.2% in 2019 to 2.5% and 4.2% in 2020 and 2021 respectively, while those of methamphetamine, alcohol, and tobacco use had decreased. Trends in past-year drinking/eating cannabis products had increased, especially among the middle age group (40–49 years) from 2.1% (95% confidence interval (CI): 1.3, 3.1) in 2019 and 1.1% (95% CI: 0.6, 1.9) in 2020 to 3.8% (95% CI: 2.8, 5.0) in 2021. The younger population aged 18–19 had an increase in cannabis smoking from 0.9% (95% CI: 0.1, 3.3) in 2019 to 2.0% (95% CI: 0.5, 5.1) and 2.2% (95% CI: 0.7, 5.1) in 2020 and 2021 respectively. Symptoms of cannabis use disorder among cannabis users increased from 2019 to 2020 and then reversed afterwards in 2021. Thais had greater health knowledge about the benefits and harms of cannabis and had attitudes toward more harm of cannabis in 2021; however, 35.6% or a third of the sample in 2021 truly believed that cannabis was a cure for cancer, and 23.2% or one-fourth were uncertain or did not believe that cannabis was addictive.

**Conclusions:**

Although most of the substances had a lower prevalence of use during the COVID-19 pandemic in Thailand, cannabis had a higher use after legalization. Thai youth had a growing trend to smoke cannabis.

## Introduction

Cannabis is the most widely used drug in the world, with an estimated 4% of the global population aged 15–64 years in 2019 having used cannabis in the past year [1]. Currently, Canada and Uruguay are the only two countries in the world that allow the nationwide sale of non-medical cannabis for adult recreational use [2]. Although cannabis use trends have increased by 18% between 2010 and 2019 and the highest annual prevalence of cannabis use was in North America (14.5%), Australia and New Zealand (12.1%), and West and Central Africa (9.4%), a lower prevalence of cannabis use has been observed in Asian countries (2.0%) [1]. For example, National Household Surveys on Drug Use in Thailand, a low- and middle-income country located in South East Asia, estimated that the Thai population aged 12 to 65 years with annual cannabis use was only approximately 1% in the past decade [3, 4].

Thailand first began developing its drug laws in 1922 when the Drug Act was drafted and used with four adaptations through 1978 [5]. Thailand then issued another Drug Act in 1979 that had been used for more than 40 years with several adaptations [6]. One of landmarks of the Thai drug policy was the declaration of war on drugs in 2003 with a harsh measure against people involved with drugs, which resulted in overcrowding in prisons by inmates sentenced for drug crimes [7]. Although still using the Drug Act issued in 1979, Thailand received the announcement Number 108/57 from the National Council for Peace and Order (NCPO) in 2014 to “decriminalize” all drug use. People who were arrested for drug use had the option of receiving treatment without penalty or criminal record [8]. In 2016, the decriminalization of drug use in Thailand received more attention, in part due to key people working in the area of drugs control in Thailand attending the Special Session of the General Assembly of the United Nations (UNGASS) on the global drug problem and began to draft the new drug act for Thailand [9]. At that time, academic and social movements also worked to support the acceptance of cannabis use for medical purposes in Thailand [10]. In 2019, the 1979 Thai Drug Act (issue 7) allowed cannabis for medical use for the first time in modern Thai history since the Cannabis Act and the Drug Act were issued in 1934 and 1979 respectively [11].

The change in drug policy towards more liberal measures against drugs in Thailand began in 2016, when the new Drug Act was first drafted as a measure to decriminalize and reduce the prison occupancy of people who used drugs in Thailand. The Drug Act was then formally issued in 2019 for the first time in modern Thai history to allow the use of cannabis for medical purposes and was renewed again in 2021 to fully exonerate those in prison for drug possession with amounts lower than the allowed limit or use charges [12]. Thailand has allowed the use of cannabis for medical purposes since 2019, when home cultivation, personal production, and sale were not allowed at the time, and is now the first country in Asia to allow the sale of cannabis, home cultivation, and household use for recreational purposes nationwide in June 2022 [13]. Parts of the cannabis plant, such as the leaves that contain low levels of delta-9-tetrahydrocannabinol (delta-9-THC), have been legalized for recreational use in Thailand for the purpose of stimulating the previously stagnant Thai economy [14] through retail sales and household use since February 2021 [15], then in 2022, Thailand removed all parts of the cannabis plant from the list of illegal drugs. At the time, only the consumable packaging product was controlled under Thai food laws [16] for the limit of THC concentration per package [15]. The recommendation on food premises was later announced regarding the preparation of cannabis-based foods for their customers [17]. Studies in the academic sector showed that the level of THC in food and drink from some Thai stores was higher than the recommended limit for consumable packaging products [18]. The Cannabis Act was drafted and is awaiting review and approval from the parliament without specific dates to be issued yet, resulting in no direct regulation on the ‘personal’ production, possession, or use of cannabis plants in Thailand since the delisting of cannabis from the narcotic drug list on June 9, 2022. However, due to the hugh public concern, cannabis was then listed as a controlled herb under the Traditional Medicine Wisdom Protection Health Act in June 2022 through the warnings for it not to be used or purchased by the young population younger than 20 years and pregnant women.

The purpose of the study is to report trends in cannabis and other substance use, cannabis use disorder, and the attitude towards cannabis use of the Thai population before and after legalization of cannabis use. Data from the annual surveys conducted in the last two months of each year in three years from 2019 to 2021 were used in the study. The study covered the period before and after legalization of recreational cannabis to use and purchase cannabis leaves in February 2021 and the movement to legalize the entire cannabis plant in December 2021, although it did not yet cover the period of availability for legal purchase of the entire cannabis plant for recreational use in June 2022.

Since surveys were conducted before and during the COVID-19 pandemic, such effects of the pandemic cannot be disentangled from the change in cannabis use due to policy changes. The history of Thai drug policy to the time of legalization of cannabis and drug decriminalization was also discussed. The results shown in this study may be useful for other developing countries with the same region and context as Thailand.

## Methods

### Sample population

Data on the use of cannabis and other substances, cannabis use disorder, and the attitude towards cannabis of the Thai population aged 18–65 years in 2019 (n = 5,002), 2020 (n = 5,389) and 2021 (n = 5,669) were obtained from annual nationwide surveys of the Centre for Addiction Studies (CADS) conducted by the Research Centre for Social and Business Development (SAB). The same provinces were surveyed over three years. Mixed method sampling was used in all three years of annual surveys to represent the Thai population. In brief, stratified multi-stage sampling was first used to identify areas including the Bangkok metropolis, the central, north, northeast, and south of Thailand as stratum and office regions of the Office of the Narcotic Control Board as sub-stratum. Then the cumulative systematic sampling, a modified systematic sampling method by adding the cumulative frequency of population size as a database for the sampling, was used to sampling the areas of each substratum based on the population in each area. The probability proportional to size sampling method was used to sample a certain number of populations in each region, province, and village by designating the proportion of the sample to be consistent with the population size in each area. Then, systematic sampling was used to select the households in each village. The inclusion criteria for the surveys were Thai citizens aged 18 to 65 years who lived in the household for at least 3 months and were able to communicate in Thai. If more than one person in a household were eligible to be included and matched with the demographics (e.g., age, sex) of the population in the region, only one was selected by convenience sampling. The response rates were approximately 80–85% during the three years of the survey.

The field research assistants who collected the data comprised 10 teams with 4 persons in each team. All research assistants were trained to complete the questionnaire before entering the fields to conduct the face-to-face interview and instructed to check for missing data and fix them with the respondents. Global Positioning System (GPS) verification was used to monitor the accuracy of the selected household to be included in the study. Informed consent by action was applied. In summary, field interviewers distributed the study participant information sheet and described the study details to participants. If eligible participants agreed to participate, the action of answering the questionnaire questions implied that they gave their consent to participate in the study. The study was approved by the Institutional Review Board of the Faculty of Medicine of Chulalongkorn University (Med Chula IRB No. 737/64). Only youths aged 18–19 were recruited in this study because the last survey in 2021 included only that age range as requested by the IRB to avoid requesting consent from parents.

### Measurement

The questionnaires were divided into four sections including (1) demographics (age, sex, marital status, level of education, employment, monthly income, areas of living) (2) variables of use of cannabis and other substances, (3) cannabis use disorder, and (4) attitude towards cannabis. Only questions that were repeatedly asked for at least two of the three years of annual surveys conducted between the years 2019 and 2021 were included in the study. The questionnaires were paper-based and took about 20–25 minutes to complete.

Substance use variables were the main key outcomes of the study. Recreational use of cannabis and other substances in lifetime, past year, and past month intervals were asked, as well as routes of administration (e.g., smoking or oral use). Eleven symptoms of cannabis use disorder (the Fifth Edition of the Diagnostic and Statistical Manual of Mental Disorders (DSM-5)) [19] were obtained by using the Thai Substance Use Disorder Screening Test (SUDST; Cronbach’s alpha coefficient = 0.8, Cohen’s kappa = 0.5, concurrent validity with clinical diagnosis and Mini International Neuropsychiatric Interview (M.I.N.I.) using a contingency coefficient with p-values < 0.001) [20]. The sum of the number of symptoms was used to identify people with a mild (2–3 symptoms), moderate (4–5 symptoms), and severe (6–11 symptoms) diagnosis of cannabis use disorder.

Attitudes toward cannabis use were measured using the question “Do you agree to remove cannabis from the list of illegal drugs?”. The respondents were also asked to determine whether they believed in the benefits and harmful effects of cannabis use, including 12 health conditions (Cronbach’s alpha coefficient = 0.875 and 0.824, respectively) that are believed to be treatable with cannabis (e.g., muscle rigidity, retractable epilepsy, neuropathic pain, chemotherapy-induced nausea/vomiting, cancer (a cure), Alzheimer’s disease, Parkinson’s disease, migraine, insomnia, glaucoma, skin diseases, anorexia) and 12 adverse or harmful effects of cannabis on physical and mental health (i.e., deteriorating physical health, addiction / dependency, hallucination, impaired vehicle operation, impaired judgement or motor function, impaired intellectual function, impaired immune function, respiratory disease, sexual dysfunction, depression/suicide, myocardial infarction, cerebral vascular disease). The number of participants’ responses yes to each health benefit and harm was used to indirectly reflect a positive and negative attitude toward cannabis use.

### Statistical analysis

Data were weighted according to demographic characteristics (e.g., age, sex, geographic region) of the Thai population. Sampling weights were calculated for cannabis and other substance use and compared between the three years of annual surveys by using the adjusted Pearson’s Chi-square test in the Complex Samples package in SPSS. Demographic data, cannabis use disorder, and attitude towards cannabis between years were compared using the Chi-square test. A Bayesian comparison was used to compare the prevalence of the oral route of administration of cannabis and cannabis smoking in different age groups between the years 2019 and 2020 and between 2020 and 2021. Specifically, the mean differences in prevalence between age groups were analyzed. Bayesian related sample inference [21] was used to specify the years of investigation in pairs. The posterior mean identifies the difference in mean prevalence between the years 2019–2020 and 2020–2021. Regarding the attitude towards cannabis, the average numbers of health conditions that were believed to be treatable and caused by cannabis between the years 2019 and 2021 were compared using an independent samples t-test. IBM SPSS Statistics Version 28.0.0.0 was used for the analysis.

## Results

The majority of the participants aged 30 and older (75.4-76.6%), were female (51.5-52.4%) and married (63.8-67.6%), finished school higher than the junior high level (50.9–53.2%) and lived outside of the municipality (59.3-61.5%). One-third (33.6-33.7%) of the sample lived in the northeastern region of Thailand and one-fourth (21.3-24.2%) worked as labor workers. About 38.3-44.6% had monthly income in the range of 10,001–20,000 Thai baht (~ 300–700 US dollars). In the survey years 2020 and/or 2021, a higher proportion lived outside the municipality (p = 0.040), were single, unemployed, and had lower income (p < 0.001) than the survey in 2019. Sex, age, and level of education did not differ significantly between the years of the survey (p > 0.05) (Table 1).

**Table 1:** Demographic data of the Thai population aged 18 to 65 years in annual surveys on substance use behaviors between 2019 and 2021

	2019 (n = 5,002)		2020 (n = 5,389)		2021 (n = 5,669)		*X* ^2^_df_	P-values
	n	(%)^a^	n	(%)^a^	n	(%)^a^		
**Age (years)**								
< 20	220	(4.4)	201	(3.7)	230	(4.1)	7.855_4_	0.097
20–29	952	(19.0)	1116	(20.7)	1166	(20.6)		
≥ 30	3830	(76.6)	4072	(75.6)	4273	(75.4)		
**Sex**								
Male	2383	(47.6)	2597	(48.2)	2750	(48.5)	0.814_2_	0.666
Female	2619	(52.4)	2792	(51.8)	2919	(51.5)		
**Regions**								
Bangkok metropolis	700	(14.0)	782	(14.5)	816	(14.4)	4.890_8_	0.769
Central	990	(19.8)	1,093	(20.3)	1,159	(20.4)		
North	949	(19.0)	952	(17.7)	999	(17.6)		
Northeast	1,686	(33.7)	1,811	(33.6)	1,906	(33.6)		
South	677	(13.5)	751	(13.9)	789	(13.9)		
**Area of living**								
Bangkok or municipality	2,037	(40.7)	2,085	(38.7)	2,185	(38.5)	6.449_2_	0.040*
Outside the municipality	2,965	(59.3)	3,304	(61.3)	3,484	(61.5)		
**Level of education**								
< High school junior	1330	(26.6)	1363	(25.3)	1364	(24.1)	9.246_4_	0.055
High school junior	1122	(22.4)	1223	(22.7)	1286	(22.7)		
> High school junior	2546	(50.9)	2793	(51.9)	3008	(53.2)		
**Marital status**								
Single	1,335	(26.7)	1,408	(26.1)	1,624	(28.7)	58.282_4_	< 0.001**
Married	3,382	(67.6)	3,477	(64.6)	3,615	(63.8)		
Divorced/widow/separated	285	(5.7)	501	(9.3)	426	(7.5)		
**Occupation**								
Unemployed	76	(1.5)	75	(1.4)	172	(3.0)	153.073_10_	< 0.001**
Retired / housework	358	(7.2)	304	(5.6)	409	(7.2)		
Students	336	(6.7)	407	(7.6)	403	(7.1)		
Labor	1102	(22.0)	1148	(21.3)	1370	(24.2)		
Agriculture / fishery	238	(4.8)	475	(8.8)	363	(6.4)		
Others	2892	(57.8)	2980	(55.3)	2952	(52.1)		
**Monthly income (THB)** ^**b**^								
≤ 5,000	511	(10.2)	601	(11.2)	738	(13.0)	102.548_8_	< 0.001**
5,001–10,000	1,512	(30.2)	1,639	(30.4)	1,629	(28.7)		
10,001–20,000	2,233	(44.6)	2,179	(40.4)	2,170	(38.3)		
20,001–30,000	531	(10.6)	655	(12.2)	710	(12.5)		
> 30,000	215	(4.3)	315	(5.8)	422	(7.4)		

*p < 0.05, **p < 0.001, Chi-square test

^a^ Unweighted estimates

^b^ Thai baht

**Fig. 1:**
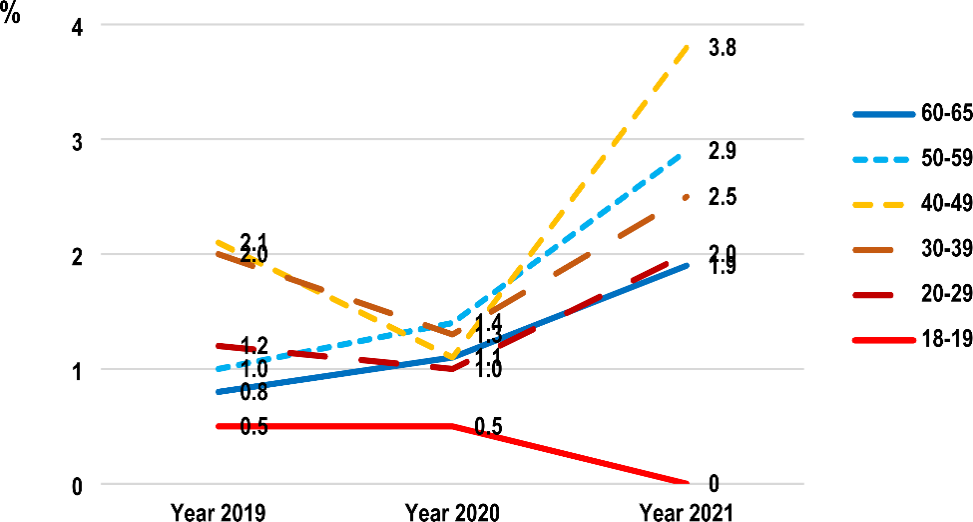
Prevalence of past-year recreational cannabis “eating or drinking” in different age groups of the Thai population aged 18–65 years across the three years between 2019 and 2021. In a Bayesian comparison of population prevalence, the posterior mean difference is -0.18 (2019–2020) with a 95% confidence interval = -1.12, 0.75 and 1.10 (2020–2021) with a 95% confidence interval = -0.65, 2.85

**Fig. 2:**
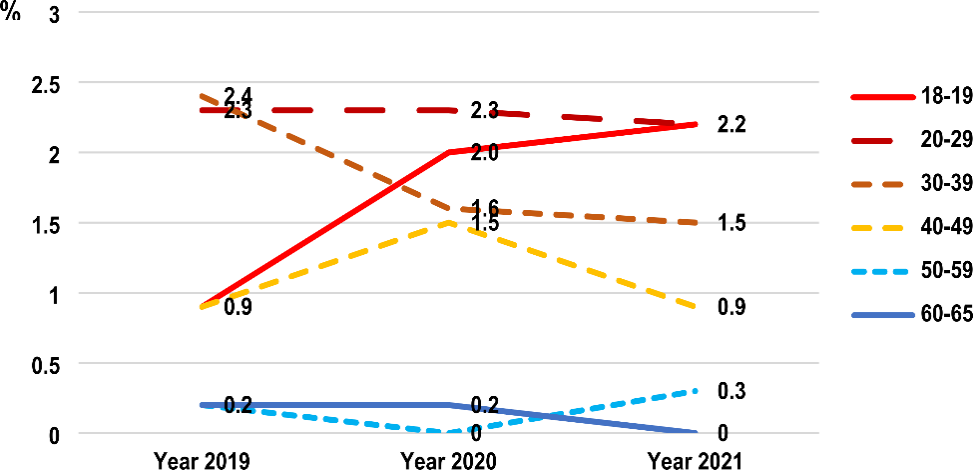
Prevalence of recreational cannabis smoking in the last year in different age groups of the Thai population aged 18–65 years between 2019 and 2021. A Bayesian comparison of population prevalence, the posterior mean difference is 0.12 (2019–2020) with 95% confidence interval = -0.99, 1.22 and − 0.08 (2020–2021) with 95% confidence interval = − 0.62, 0.45

Table 2 shows the use of cannabis and other substances during the three years. The prevalence of methamphetamine use in the past year and last month had decreased between 2020 and 2021. In contrast, the prevalence of cannabis and kratom use in the past year and last month had increased. Kratom (Mitragyna speciosa, Korth.) is an addictive plant whose leaves have been popularly consumed in countries of southeast Asia, including Thailand. However, the lifetime prevalence of cannabis use was 9.9%, 6.2% and 6.4% in 2019, 2020 and 2021 respectively. The lifetime use of legal substances, including alcohol and tobacco, had decreased (p < 0.001) but those of illegal substances, including kratom (p < 0.001) had increased from 2019 to 2021. Regarding the routes of administration, the trend of cannabis smoking in the past year had decreased (p < 0.022) whereas consumption of cannabis in edible food products or beverages in the past year increased, but was not statistically significant (p > 0.05) (Table 2), especially among the middle-aged population (40–49 years) (Fig. 1). The prevalence of oral route of cannabis administration (e.g., sublingual cannabis oil, edible cannabis such as cookies, candy, food, beverages) in the past year was 2.1% (95% confidence interval (CI): 1.3, 3.1), 1.1% (95% CI: 0.6, 1.9), and 3.8% (95% CI: 2.8, 5.0) in 2019, 2020 and 2021, respectively, in the Thai population aged 40–49 years and was 1.0% (95% CI: 0.5, 1.7), 1.4% (95% CI: 0.8, 2.3) and 2.9% (95% CI: 2.0, 4.0) in 2019, 2020 and 2021, respectively, in the Thai population aged 50–59 (Fig. 1). In contrast to the trend of cannabis smoking in the past year in the total population, the younger population aged 18–19 had a higher trend of cannabis smoking from 0.9% in 2019 (95% CI: 0.1, 3.3) to 2.0% (95% CI: 0.5, 5.1) and 2.2% (95% CI: 0.7, 5.1) in 2020 and 2021 respectively (Fig. 2).

**Table 2:** Prevalence of use of legal and illegal substances in life, past year, and last month of the Thai population aged 18–65 between 2019 and 2021

	2019 (n = 5002)					2020 (n = 5389)					2021 (n = 5669)					Adjusted F_df1, df2_	P-values
	n	%^a^	Preva- lence (%)^b^	95% confidence intervals	Weighted number	n	%^a^	Preva- lence (%)^b^	95% confidence intervals	Weighted number	n	%^a^	Preva- lence (%)^b^	95% confidence intervals	Weighted number		
**Lifetime**																	
Tobacco	2,081	41.6	42.1	42.09–42.12	20,772,536	1,786	33.1	33.5	33.47–33.49	14,982,175	1,623	28.6	28.9	28.87–28.90	12,878,374	13.1_1.5, 35.2_	< 0.001***
Alcohol	3,263	65.2	65.6	65.62–65.65	32,378,450	3,000	55.7	56.0	55.95–55.98	25,045,434	2,729	48.1	48.3	48.26–48.29	21,523,662	12.8_1.5, 37.1_	< 0.001***
Cannabis	496	9.9	10.1	10.10-10.11	4,984,683	334	6.2	6.2	6.24–6.25	2,794,914	361	6.4	6.4	6.40–6.42	2,857,484	9.2_1.6, 39.0_	0.001**
Kratom leaves	303	6.1	6.20	6.19–6.20	3,057,364	267	5.0	4.9	4.89–4.90	2,190,528	729	12.9	12.8	12.81–12.83	5,718,039	14.4_1.7, 39.8_	< 0.001***
Kratom cocktail	178	3.6	3.68	3.67–3.68	1,814,226	66	1.2	1.2	1.25–1.26	560,770	263	4.6	4.7	4.67–4.68	2,084,922	9.2_1.4, 34.5_	< 0.001***
Methamphetamine	75	1.5	1.55	1.55–1.56	766,710	97	1.8	1.8	1.82–1.83	816,727	134	2.4	2.4	2.37–2.38	1,059,023	1.3_1.8, 42.6_	0.269
Inhalants	10	0.2	0.22	0.22–0.22	110,251	15	0.3	0.3	0.28–0.28	124,388	16	0.3	0.3	0.28–0.29	127,322	0.1_1.6, 37.9_	0.831
Others^c^	13	0.3	0.29	0.29–0.29	143,218	12	0.2	0.2	0.22–0.22	98,572	16	0.3	0.3	0.29–0.29	127,980	0.4_1.3, 30.5_	0.601
**Past-year**																	
Tobacco	1,401	28.0	28.5	28.53–28.56	14,081,141	1,233	22.9	23.2	23.14–23.17	10,362,083	1,244	21.9	22.1	22.09–22.12	9,857,004	5.7_1.5, 36.4_	0.012*
Alcohol	2,526	50.5	51.0	51.02–51.05	25,175,616	2,170	40.3	40.6	40.59–40.62	18,169,572	2,080	36.7	36.8	36.75–36.78	16,392,140	15.2_1.7, 39.9_	< 0.001***
Cannabis all routes	112	2.2	2.3	2.29–2.30	1,131,776	133	2.5	2.5	2.49–2.50	1,116,346	240	4.2	4.3	4.25–4.27	1,899,426	3.1_1.9, 45.1_	0.058
Smoking^d^	60	53.6	51.8	51.68–51.86	615,968	68	51.1	51.2	51.19–51.38	572,511	64	26.7	26.6	26.57–26.69	505,787	4.3_1.8, 44.1_	0.022*
Oral use^d^	67	59.8	59.5	59.44–59.61	708,204	63	47.4	47.2	47.08–47.27	526,609	147	61.3	61.2	61.13–61.26	1,162,359	0.9_1.8, 44.1_	0.390
Kratom leaves	126	2.5	2.6	2.61–2.61	1,287,351	150	2.8	2.7	2.72–2.73	1,219,896	642	11.3	11.3	11.28–11.3	5,032,634	26.2_1.7, 40.0_	< 0.001***
Kratom cocktail	40	0.8	0.9	0.87–0.88	431,041	35	0.6	0.7	0.66–0.67	296,546	199	3.5	3.6	3.55–3.56	1,585,543	22.4_1.3, 30.5_	< 0.001***
Methamphetamine	4	0.1	0.1	0.08–0.09	41,756	29	0.5	0.5	0.55–0.55	247,252	7	0.1	0.1	0.13–0.13	57,109	12.9_2.0, 47.0_	< 0.001***
**Past-month**																	
Tobacco	-	-	-	-	-	1,189	22.1	22.3	22.31–22.34	9,990,206	1,227	21.6	21.8	21.79–21.82	9,722,535	0.05_1, 24_	0.826
Alcohol	-	-	-	-	-	2,043	37.9	38.2	38.2-38.23	17,103,039	1,800	31.8	31.8	31.83–31.86	14,200,474	8.1_1, 24_	0.009**
Cannabis all routes	-	-	-	-	-	105	1.9	2.0	1.97–1.98	882,506	190	3.4	3.4	3.38–3.40	1,511,512	5.0_1, 24_	0.035*
Kratom leaves	-	-	-	-	-	142	2.6	2.6	2.57–2.58	1,153,816	606	10.7	10.7	10.65–10.66	4,750,808	53.7_1, 24_	< 0.001***
Kratom cocktail	-	-	-	-	-	31	0.6	0.6	0.58–0.59	262,382	184	3.2	3.3	3.29–3.30	1,467,382	71.3_1, 24_	< 0.001***
Methamphetamine	-	-	-	-	-	18	0.3	0.3	0.34–0.35	154,055	6	0.1	0.1	0.11–0.11	49,166	4.9_1, 24_	0.037*

*p < 0.05, **p < 0.01, ***p < 0.001, Adjusted Pearson Chi-square test

^a^ Unweighted estimates

^b^ Weighted estimates

^c^ Opium, morphine, heroin, ecstasy, ketamine, cocaine

^d^ Conditional estimates among recreational cannabis users in the past year

Regarding cannabis use disorder, trends were mixed between years 2019 and 2020 and between years 2020 and 2021 (Table 3). Almost all symptoms of cannabis use disorder based on the DSM-5 criteria increased between 2019 and 2020 then reversed afterwards. Likewise, Thai cannabis users had a higher prevalence of cannabis use disorder (i.e., harmful use or dependence on cannabis) in 2020 compared to 2019 after the medical cannabis law was imposed. However, a lower prevalence was observed in 2021 (Table 3).

**Table 3:** Cannabis use disorder between the years 2019 and 2021 in the Thai population aged 18–65 years who used cannabis in the past 12 months

	2019 (n = 112)		2020 (n = 133)		2021 (n = 165)		*X* ^2^	P-values
	n	%^a^	n	%^a^	n	%^a^		
**Symptoms of DSM-5 cannabis use disorder**								
1. Used more than intended	11	(9.8)	26	(19.5)	12	(7.3)	11.211	0.004**
2. Unable to stop or cut down	9	(8.0)	23	(17.3)	6	(3.6)	16.611	< 0.001***
3. Spent a lot of time on cannabis.	4	(3.6)	11	(8.3)	4	(2.4)	6.089	0.048*
4. Craving	10	(8.9)	15	(11.3)	9	(5.5)	3.366	0.186
5. Impaired function	16	(14.3)	16	(12.0)	4	(2.4)	14.314	0.001**
6. Still using cannabis despite having a relationship problem	19	(17.0)	35	(26.3)	5	(3.0)	33.240	< 0.001***
7. Reduced other recreational activities	8	(7.1)	17	(12.8)	5	(3.0)	10.333	0.006**
8. Cannabis used in a risky situation	16	(14.3)	33	(24.8)	15	(9.1)	14.022	0.001**
9. Still using cannabis despite impaired physical or mental health	22	(19.6)	36	(27.1)	16	(9.7)	15.288	< 0.001***
10. Tolerance	6	(5.4)	13	(9.8)	4	(2.4)	7.532	0.023*
11. Withdrawal	2	(1.8)	11	(8.3)	5	(3.0)	7.308	0.026*
**DSM-5 cannabis use disorder**								
- No (0–1 symptom)	79	(70.5)	80	(60.2)	144	(87.3)	38.506	< 0.001***
- Mild (2–3 symptoms)	16	(14.3)	29	(21.8)	13	(7.9)		
- Moderate (4–5 symptoms)	15	(13.4)	12	(9.0)	5	(3.0)		
- Severe (6–11 symptoms)	2	(1.8)	12	(9.0)	3	(1.8)		

*p < 0.05, **p < 0.01, ***p < 0.001, Chi-square test

^a^ Unweighted estimates

Table 4 shows the attitude of the Thai population towards cannabis. Although about half of Thais agreed with the idea of removing cannabis from the list of illegal drugs in 2019, the Thai population was more uncertain in 2020 (p < 0.001). Furthermore, Thais had a higher level of health literacy about the benefits and harms of cannabis in 2021 compared to the year 2019. For example, although up to 39% of the sample thought that cannabis could definitely be used to cure cancer in 2019, the percentage decreased to 35.6% in 2021 (p < 0.001). Similarly, Thais realized more about the harm caused by cannabis to their physical and mental health (p < 0.001) (Table 4).

**Table 4:** Attitude towards cannabis in the Thai population aged 18 to 65 between 2019 and 2021

	Prior		Later		*X*^2^ or T	P-values
	n	%^a^	n	%^a^		
**Opinion on removing cannabis from the narcotics list** ^**b**^						
Agree	2527	(50.5)	2337	(43.4)	1078.177	< 0.001***
Not agree	1724	(34.5)	803	(14.9)		
Unsure	751	(15.0)	2249	(41.7)		
**Believing and very sure that cannabis can cure the following symptoms / diseases** ^**c**^						
1. Cancer	1977	(39.5)	2018	(35.6)	17.497	< 0.001***
2. Chemotherapy-induced nausea/vomiting	608	(12.2)	1491	(26.3)	336.524	< 0.001***
3. Insomnia	1540	(30.8)	1688	(29.8)	1.289	0.256
4. Muscle rigidity	1228	(24.6)	1394	(24.6)	0.002	0.962
5. Neuropathic pain	1051	(21.0)	1690	(29.8)	107.797	< 0.001***
6. Migraine	871	(17.4)	1206	(21.3)	25.265	< 0.001***
7. Retractable epilepsy	693	(13.9)	1301	(22.9)	144.663	< 0.001***
8. Alzheimer’s disease	607	(12.1)	995	(17.6)	61.103	< 0.001***
9. Parkinson’s disease	762	(15.2)	847	(14.9)	0.178	0.673
10. Skin diseases	419	(8.4)	513	(9.0)	1.508	0.219
11. Glaucoma	227	(4.5)	465	(8.2)	58.837	< 0.001***
**Believing and very sure that cannabis can cause the following symptoms / diseases** ^**c**^						
1. Deteriorating physical health	3128	(62.5)	4858	(85.7)	756.879	< 0.001***
2. Cannabis addiction/dependence	3615	(72.3)	4353	(76.8)	28.639	< 0.001***
3. Hallucination	2560	(51.2)	4006	(70.7)	426.250	< 0.001***
4. Impaired vehicle operation	2281	(45.6)	3397	(59.9)	218.888	< 0.001***
5. Impaired judgment or motor function	1851	(37.0)	3247	(57.3)	437.649	< 0.001***
6. Impaired intellectual function	1292	(25.8)	2636	(46.5)	488.048	< 0.001***
7. Impaired immune function	1461	(29.2)	3698	(65.2)	1380.880	< 0.001***
8. Lung and respiratory diseases	1385	(27.7)	3429	(60.5)	1154.428	< 0.001***
9. Sexual dysfunction	1018	(20.4)	2402	(42.4)	591.594	< 0.001***
10. Depressive disorder or suicide	895	(17.9)	2402	(42.4)	745.733	< 0.001***
11. Myocardial infarction	756	(15.1)	1710	(30.2)	338.737	< 0.001***
12. Cerebral vascular disease	746	(14.9)	1688	(29.8)	333.362	< 0.001***
**Number of diseases/symptoms believed and be sure that cannabis can treat** ^**c**^ **(min = 0, max = 11) (mean, SD)**	2.00	2.45	2.40	2.63	8.224	< 0.001^†††^
**Number of diseases/symptoms believed and very sure that cannabis can cause** ^**c**^ **(min = 0, max = 12) (mean, SD)**	4.20	3.26	6.67	3.35	38.605	< 0.001^†††^

***p < 0.001, Chi-square test

^†††^p < 0.001, unpaired t-test

^a^ Unweighted estimates

^b^ Years 2019 (n = 5,002) versus 2020 (n = 5,389)

^c^ Years 2019 (n = 5,002) versus 2021 (n = 5,669)

## Discussion

The policy on cannabis had changed rapidly in Thailand, as shown by the legalization of cannabis for recreational purposes within a few years after the medical allowance in 2019. Although lifetime cannabis use trends and other substance use trends were mixed, the study shows an increased trend in cannabis use in the last month, while the use of methamphetamine, alcohol, and tobacco in the past year and the past month had decreased. The latter results were most likely due to the COVID-19 lockdown measures when Thai authorities prohibited the sale of alcohol nationwide and advised everyone to stop drinking and smoking at the time of the pandemic to stay healthy. Cannabis consumption and cannabis use disorder were higher in 2020 than in 2019, which is the year of the passing of the medical cannabis law. The trend to smoke cannabis in the past year had increased among Thai youth. Although health literacy on the benefits and harm of cannabis had improved in 2021, up to a third of Thais still believed that cannabis could cure cancer and a quarter were still uncertain or did not believe that cannabis was addictive.

Cannabis has been a topic of debate around the world on weighing its harms versus benefits for drug policy reforms, such as in Canada [22]. However, countries in Asia, such as Thailand, did not have a high prevalence of cannabis use as in western countries [23]. The legalization of non-medical cannabis for adult recreational use was rapidly implemented between 2019 and 2022. The Free Cannabis Policy was raised as a political party’s campaign for national elections [24] at the same time as the issue of the medical cannabis law in 2019 [9, 25]. Subsequently, the campaign was changed to support the Free ‘Medical’ Cannabis Policy [26] which did not support permission for recreational purposes. The increased trend of the past-year cannabis use and cannabis use disorder between 2019 and 2021 in Thailand was consistent with previous reports of increased cannabis use and harm after the loosening of the non-medical cannabis law in the US at the state level and Canada at the federal level [27]. An increased amount of cannabis use and/or cannabis use disorder [28–30] and cannabis harm [31] were observed in the general population of the US, as well as in the subgroup of the US population with depression [32]. Similarly, hospitalizations or emergency department visits due to cannabis harms in Canada had increased after legalization of cannabis [33, 34]. Notably, research in this area within the geographical region of Thailand is limiting because recreational cannabis use is prohibited in other countries in Asia.

One of the most concerning issues is the growing trend for smoking cannabis among Thai youth. A retrospective longitudinal study of the exposure of young Americans to the cannabis policy showed an increased trend toward cannabis use later in life, especially when they were subjected to medical cannabis laws at a young age [35]. College students in states with legalization of cannabis used more cannabis than those without [36]. Increased routes / modes of cannabis administration were observed in Canadian youth in 2018 and multimodal cannabis use (e.g., smoking, vaping, eating or drinking) was associated with other substance use and depressive symptoms [37]. Furthermore, studies in Canada showed an unchanged but still high prevalence of youth cannabis use [38]. An increased trend of cannabis use during pregnancy was reported in a population-based study in Ontario, Canada [39] reflecting that young citizens might eventually be more exposed to cannabis in their prenatal stage.

Our study shows that the prevalence of cannabis use disorder in 2020 was higher than in 2019 which was the year of allowance for medical purposes, and was higher than the prevalence in 2021 before the time to allow cannabis for recreational purposes. This is consistent with the results of our study that Thai people’s health literacy about the health benefits and harms of cannabis could improve in recent years (i.e., lower proportion of Thais in 2021 compared to those in 2019 believed that cannabis can cure cancer and is not addictive). The reasons for the increased knowledge of cannabis may be due to experiences in the news reporting serious side effects of patients who used cannabis oil for medical purposes in 2019 when the medical cannabis law was issued. The government and especially folk healers and Thai traditional medicine practitioners were seen to promote the health benefits of cannabis to encourage patients to use cannabis [40, 41]. However, other professional organizations, including the Thai Medical Council, some departments of the Ministry of Public Health, and various academic sectors, tried to educate Thais about the harmful effects of cannabis and the limit for medical purposes [42–46]. This movement may be helpful for Thais to have more knowledge and health literacy about cannabis. However, a large number of Thai people still believe that cannabis can cure cancer in humans and that it is not addictive, which still allows an open opportunity to implement more knowledge on the harms and limits of the benefits of cannabis to the Thai population in the future to mitigate the effects of medical cannabis law and legalization of cannabis in Thailand.

The Thai Ministry of Public Health delisted the cannabis plant from the drug list in February 2022 and only the cannabis extract with a THC concentration higher than 0.2% by weight is still considered illegal and is on the drug list. Thais could grow cannabis plants in their homes without permission and received cannabis plants from the government free of charge [47]. Through publicizing of the health benefits of cannabis, many patients with various diseases sought to use cannabis products themselves, through traditional medical practice, or some through official services such as the Ganja clinic, opened by the Ministry of Public Health [40]. In 2019, reports of accidental incidents of Thais who used cannabis oil to treat their medical conditions from mild to severe symptoms, but then had severe side effects were observed [48]. The prevalence of cannabis use in the past year of the Thai population aged 18–65 in 2021 was up to 4% and those in 2022 was 25% [49] since cannabis food and drink can be easily accessed in department stores, street food vendors, etc. and is widely consumed by people of all age groups in Thailand [50] although warnings were made that it should not be used by the young population and pregnant women. However, a survey showed that most of the Thai population did not agree with recreational cannabis use and supported only medical cannabis use [51].

This study had several limitations, which deserves to be noted. First, the comparison of repeat cross-sectional data could not directly reflect the increase or decrease in cannabis use since the study was not a prospective cohort of the same sample. Additionally, there was no comparison group where policy did not change. It is difficult to strictly attribute differences to policy rather than variation between samples. Second, the effect on attitude and behavior usually appeared before the law was enacted, which was not covered by this study. However, our study still showed an increasing trend after implementation. Third, the stigma of drug use in Thailand might affect the responses of the survey. Participants may feel afraid to tell their own drug use, depending on the drug policy that has changed rapidly each year since 2019 in Thailand. The medical cannabis policy in 2019 was the first time in modern Thai society that allowed the use of cannabis, although only for medical purposes. Participants may feel more relaxed reporting their lifetime use of cannabis at the time. However, a decrease in the lifetime prevalence of cannabis use was observed later in the next two years. Similarly, the use of alcohol and tobacco, especially heavy use, although legal, is still a social stigma in Thai society. This might affect the response to lifetime use of alcohol and tobacco of ex-drinkers and ex-smokers in the surveys each year. Fourth, the study only covers the population aged 18 to 65 years, and not the young adolescents aged younger than 18 years, who may be more at risk for cannabis use-related harms. More studies are needed to include younger age to study the effect of cannabis legalization and medical law. Lastly, since the surveys were conducted before and during the COVID-19 pandemic, the effects of the pandemic could affect the results. However, Thailand legalized cannabis at the time of the pandemic, so the study could not be carried out otherwise. Furthermore, the decrease in trends for substances other than cannabis and kratom supported the impact of the COVID-19 pandemic on the decrease, not the increase, of substance use, while during the period of time, the use of cannabis in the past year increased slightly.

Our results were an example for other developing countries in the same region and in the same context as Thailand (i.e., low prevalence of cannabis use) to be careful when issuing a medical cannabis law or legalizing cannabis. In the near future, not only within the country, Thailand’s cannabis production can increase and invade neighboring countries, as observed in some European countries [52]. More monitoring and investigation are warranted to prevent such harmful outcomes.

## Data Availability

The datasets generated during and/or analyzed during the current study are available from the corresponding author.
